# Eyeblink Conditioning in Schizophrenia: A Critical Review

**DOI:** 10.3389/fpsyt.2015.00146

**Published:** 2015-12-18

**Authors:** Jerillyn S. Kent, Amanda R. Bolbecker, Brian F. O’Donnell, William P. Hetrick

**Affiliations:** ^1^Department of Psychological and Brain Sciences, Indiana University, Bloomington, IN, USA; ^2^Minneapolis Veterans Affairs Health Care System, Minneapolis, MN, USA; ^3^Department of Psychiatry, Indiana University School of Medicine, Indianapolis, IN, USA; ^4^Larue D. Carter Memorial Hospital, Indianapolis, IN, USA

**Keywords:** schizophrenia, cerebellum, eyeblink conditioning, associative learning, cognitive dysmetria

## Abstract

There is accruing evidence of cerebellar abnormalities in schizophrenia. The theory of cognitive dysmetria considers cerebellar dysfunction a key component of schizophrenia. Delay eyeblink conditioning (EBC), a cerebellar-dependent translational probe, is a behavioral index of cerebellar integrity. The circuitry underlying EBC has been well characterized by non-human animal research, revealing the cerebellum as the essential circuitry for the associative learning instantiated by this task. However, there have been persistent inconsistencies in EBC findings in schizophrenia. This article thoroughly reviews published studies investigating EBC in schizophrenia, with an emphasis on possible effects of antipsychotic medication and stimulus and analysis parameters on reports of EBC performance in schizophrenia. Results indicate a consistent finding of impaired EBC performance in schizophrenia, as measured by decreased rates of conditioning, and that medication or study design confounds do not account for this impairment. Results are discussed within the context of theoretical and neurochemical models of schizophrenia.

## Introduction

Growing empirical evidence suggests cerebellar abnormalities in schizophrenia. In terms of cerebellar morphology, imaging studies report reduced cerebellar volume in chronic (1–4), neuroleptic-naïve (5), adolescent (6), first-episode (7–9), and childhood-onset (10) schizophrenia [for exceptions see Ref. (11, 12)]. Postmortem studies have also found reduced size and density of Purkinje cells in schizophrenia (13–15). In addition to structure, cerebellar function has also been reported to be abnormal in schizophrenia. Functional neuroimaging studies report abnormal cerebellar activation at rest (16–18) and during cognitive tasks [Ref. (19–21); see Ref. (22) for critical review] in individuals with schizophrenia.

These structural and functional cerebellar abnormalities appear to have clinical and functional implications in schizophrenia. Specifically, cerebellar abnormalities are associated with clinical symptoms, cognitive deficits, and outcome measures in schizophrenia (3, 23–25). For example, deficits in working memory and mental flexibility correlate with cerebellar volume (26), and fronto-cerebellar metabolic abnormalities are associated with anhedonia and ambivalence (27). Moreover, increased connectivity between frontal–parietal and cerebellar regions predicts better cognitive performance in controls and individuals with schizophrenia, and individuals with schizophrenia with improved connectivity have fewer disorganization symptoms (28).

These empirical findings are often integrated into the cognitive dysmetria theory of schizophrenia, which places the cerebellum prominently in the cortico-cerebellar-thalamic-cortical circuit (CCTCC). The theory of cognitive dysmetria proposes a model of schizophrenia wherein deficits in this circuit are associated with both motor dysfunction and the clinical presentation of schizophrenia, and abnormalities in the CCTCC are believed to mediate the disordered cognition, behavior, and motor function characteristic of individuals with schizophrenia (29). A behavioral measure of cerebellar integrity, such as eyeblink conditioning (EBC), that can be administered to individuals with schizophrenia as an index of how well the cerebellum and interrelated circuits are performing is vital to the investigation of the cerebellum as a critical node in the CCTCC and locus of dysfunction in this influential theory of schizophrenia.

Eyeblink conditioning is a widely used measure of cerebellar-dependent associative learning. In the delay form of this task, a conditioned stimulus (e.g., brief tone) is paired, and co-terminates, with an unconditioned stimulus (e.g., air puff to the eye) that elicits an unconditioned response (e.g., eyeblink). Over the course of repeated paired presentations, a conditioned eyeblink response (CR) occurs in response to the tone and preceding the onset of the unconditioned stimulus. EBC is used in the study of clinical disorders such as schizophrenia and autism as well as aging for several reasons. First, the neural circuit underlying EBC has been well-characterized in non-human animals, with the specific brain stem nuclei associated with both stimulus encoding and motor output remarkably well-understood [see Ref. (30), for review]. Furthermore, the neural plasticity underlying standard delay EBC has been localized to the ipsilateral dorsal lateral anterior interpositus nucleus, and specific areas of the cerebellar cortex involved with timing and gain control of the conditioned response have also been identified [again see Ref. (30), for review]. Second, the conditioned response that develops over the course of delay EBC is well-preserved across species including rodents [e.g., Ref. (31, 32)], rabbits [e.g., Ref. (33)], cats [e.g., Ref. (34)], and humans [e.g., Ref. (35)], making EBC a widely used translational probe of cerebellar function. Finally, the associative learning induced by EBC is a non-declarative form of learning that occurs outside of intention and conscious awareness (35). Because performance on EBC is not dependent on higher-order cognitive function or the ability to follow complex instructions, it can be studied in individuals across a variety of ages and clinical presentations.

Importantly, the robust identification of cerebellar circuitry underlying delay EBC in non-human species is remarkably consistent with human EBC findings. Such evidence has emerged from studies involving patients with cerebellar lesions, dual-task interference, transcranial direct current stimulation (tDCS), and functional brain imaging. Specifically, individuals with cerebellar strokes demonstrate impairments in delay EBC performance (36–38). In addition, studies have demonstrated a significant relationship between performance on delay EBC and cerebellar-dependent timed interval tapping (39) as well as dual-task interference during simultaneous delay EBC and timed interval tapping (40) in non-psychiatric controls. tDCS applied to the cerebellum during acquisition has been shown to modify delay EBC performance (41). Finally, human brain imaging studies investigating the neural substrates of EBC converge with the lesion and dual-task studies described above, as well as further localize the site of EBC learning-related plasticity in humans. Specifically, positron emission tomography (PET) studies have revealed changes in cerebellar activation during EBC (42–46), and functional magnetic resonance imaging (fMRI) BOLD activation changes in the cerebellum are consistently reported during EBC (47–50).

In the first published review of EBC studies and schizophrenia (51), the author concluded that overall the EBC findings were inconclusive and any observed EBC deficits may be accounted for by antipsychotic medication administration. Lubow (51) called for an explicit comparison between medicated and non-medicated individuals with schizophrenia. In addition, concerns were raised about drawing firm conclusions regarding EBC impairment in schizophrenia due to inconsistencies in the analysis of EBC (i.e., whether or not studies accounted for alpha responses and spontaneous blink rate), possible group differences in processing and encoding EBC stimuli, the notorious heterogeneity present in the diagnostic category of schizophrenia, and the small sample sizes and disproportionate number of male individuals with schizophrenia reported in the literature (51).

Two subsequent brief reviews have appeared as subsections in two recently published articles, one reviewing EBC performance across many neurodevelopmental disorders (52) and another reviewing cerebellar-related motor dysfunction in schizophrenia and high-risk populations (53). The authors of both brief reviews largely emphasized the emerging pattern of abnormal EBC performance in schizophrenia, citing the large sample sizes and the persistent deficit in EBC performance in an unmedicated subsample reported in studies published after Lubow’s (51) review (52), as well as even more recent studies of EBC impairment in individuals with schizotypal personality disorder, first-degree relatives of individuals with schizophrenia, and individuals with schizophrenia who are medication-free for a period of several weeks (53). However, both groups also acknowledged the possible role of antipsychotic medication and methodological variability in the inconsistent findings across studies (52, 53).

Importantly, since the publication of Lubow’s (51) initial review of nine articles, six additional studies have been published examining EBC in the schizophrenia spectrum. These six studies account for 48% of all individuals in the schizophrenia spectrum that have participated in delay EBC studies, nearly doubling the number of participants in the schizophrenia spectrum that have been studied since Lubow’s (51) review. However, questions still persist regarding the source of inconsistency in the literature examining EBC in schizophrenia, specifically related to the potential effects of antipsychotic medication and heterogeneity in methodology.

The purpose of the present review was to conduct a thorough and integrative review of published studies of EBC in the schizophrenia spectrum. Given Lubow’s (51) findings and cautions as well as the conclusions of Reeb-Sutherland and Fox (52) and Bernard and Mittal (53), special attention was paid to (1) evidence of antipsychotic medication effects, (2) inconsistencies between studies in and any systematic effects of stimulus and analysis parameters, and (3) differences in sample size and sample characteristics. Finally, the findings of this review are interpreted within the context of existing models of schizophrenia.

## Method

Tables 1–5 catalog 15 studies examining EBC in individuals with schizophrenia. These studies were first identified using Lubow’s existing review of EBC in schizophrenia. Studies examining EBC in the schizophrenia spectrum published subsequent to this review were identified using PubMed, a resource of the National Center for Biotechnology Information (NCBI), at the National Institutes of Health’s (NIH) U.S. National Library of Medicine (NLM).

**Table 1:** **Sample characteristics for studies of EBC in schizophrenia**.

Study	Samples			Diagnosis	Age matched?	Antipsychotic medication status (SZ spectrum groups)^a^
	*N*	Age	% Male	
Taylor and Spence (54)	42	N/A	N/A	“Psychotic”	No	N/A
	74	N/A	N/A	“Neurotic”		

O’Connor and Rawnsley (55)	20	47.2 (4.94)	100	Paranoid SZ	No	N/A
	20	41.5 (5.84)	100	Non-paranoid SZ		
	20	39.4 (12.51)	100	Control		

Spain (56)	54	40.6	59.3	Schizophrenia	No	All but 10 “were receiving some form of drug treatment”
	24	N/A	50	Control		

Sears et al. (57)	15	32.8 (9.8)	73.3	DSM-IV schizophrenia	No	Unmedicated for 3 weeks
	15	31.3 (7.2)	73.3	Control		

Hofer et al. (58)	24	30.3 (9.0)	87.5	DSM-IV schizophrenia	Yes	18 participants on atypical antipsychotics, 6 on typical antipsychotics
	20	30.9 (8.9)	85	Control		

Stevens et al. (59)	25	28.8 (6.5)	56	DSM-IV schizophrenia	Yes	Treated ≥14 days with stable dose of olanzapine
	25	31.1 (6.8)	56	DSM-IV schizophrenia		Taking stable dose of “classic neuroleptics” ≥14 days
	25	27.3 (5.6)	52	Control		

Marenco et al. (60)					
Trace	10	31.8 (8.7)	N/A	DSM-IV schizophrenia or schizoaffective disorder	Yes (±3 years)	2 participants unmedicated for ≥3 weeks; others taking antipsychotics
	10	33.7 (7.7)	N/A	Control		
Delay	10	41.8 (9.7)	N/A	DSM-IV schizophrenia or schizoaffective disorder	Yes (±3 years)	3 participants unmedicated for ≥3 weeks; others taking antipsychotics
	10	41.9 (9.4)	N/A	Control		

Brown et al. (61)	13	42 (9.56)	53.8	DSM-IV schizophrenia or schizoaffective disorder	Yes	9 participants on atypical antipsychotics, 1 on typical, 3 on both
	13	40.2 (9.0)	53.8	Control		

Edwards et al. (62)	10	40 (6.77)	60	DSM-IV schizophrenia	Yes	1 participant taking typical antipsychotics, 2 taking atypical antipsychotics, 1 taking both; 5 participants not taking antipsychotics
	8	43.5 (6.2)	62.5	Control		

Bolbecker et al. (63)	62	39.8 (9.54)	62.9	DSM-IV schizophrenia	Yes (±2 years)	12 participants unmedicated, 34 on atypical antipsychotics, 7 on typical, 9 on both
	62	39.9 (9.99)	48.4	Control		
Bolbecker et al. (64)	55	41.1 (11.1)	60	DSM-IV schizophrenia spectrum disorders	Yes (±2 years)	13 unmedicated, 36 taking atypical antipsychotics, 16 taking typical antipsychotics
	55	40.9 (11.3)	47.3	Control		

Forsyth et al. (65)	18	37.7 (9.43)	55.6	DSM-IV schizophrenia	Yes	3 SZ not taking medication at time of testing, 14 on antipsychotics; 3 SPD taking antidepressants, others unmedicated
	18	38.1 (9.87)	55.6	DSM-IV SPD		
	18	37.9 (9.85)	55.6	Control		

Parker et al. (66)	20	28.2 (9.24)	61.1	DSM-IV schizophrenia	No	5 neuroleptic-naïve, all others medication-free for 3 weeks
	20	29.2 (9.22)	50	Control		

Bolbecker et al. (67)	18	36 (12)	72.2	DSM-IV schizophrenia or schizoaffective disorder	Yes (±3 years) for each triad	2 SZ unmedicated, 13 taking atypical antipsychotics, 3 taking typical antipsychotics; 2 relatives taking antidepressants, others unmedicated
	18	35.9 (13)	38.9	Confirmed first-degree relative		
	18	36.8 (13)	44.4	Control		

Coesmans et al. (68)	38	23.9 (range = 18–35)	100	DSM-IV schizophrenia	Yes	13 antipsychotic-free (6 antipsychotic-naïve, 7 antipsychotic-free for ≥4 weeks), 9 taking atypical antipsychotics, 16 taking typical antipsychotics
	26	24.6 (range = 18–31)	N/A	Control		

*^a^Given the relevance of antipsychotic medication to motor abnormalities, we report here antipsychotic medication status specifically and not other psychotropic medications, except in the case of intermediate spectrum participants*.

**Table 2:** **EBC paradigms and measurement techniques for studies of EBC in schizophrenia**.

Study	Procedure type	Method of eyeblink activity measurement
Taylor and Spence (54)	Single-cue visual delay EBC	Microtorque potentiometer mounted to lever attached to upper eyelid and polygraph recording eyelid position (69)
O’Connor and Rawnsley (55)	Single-cue auditory delay EBC	Light source and photoelectric cell
Spain (56)	Combined visual and auditory delay (presented as 50 trials of each, order counterbalanced)	
Auditory	Auditory delay EBC	Electrodes placed above and below the eye; similar to electrooculography
Visual	Visual delay EBC	Electrodes placed above and below the eye; similar to electrooculography
Sears et al. (57)	Single-cue auditory delay EBC	Infrared photobeam
Hofer et al. (58)	Delay eyelid conditional discrimination learning	Photocell that measured “area of the eye covered by the eyelid” (via “light reflected from the cornea”)
Stevens et al. (59)	Discrimination auditory delay EBC	–
Marenco et al. (60)		
Trace	Single-cue auditory trace EBC	“Potentiometer attached to the eyelid”
Delay	Single-cue auditory delay EBC	“Potentiometer attached to the eyelid”
Brown et al. (61)	Single-cue auditory delay EBC	EMG electrodes
Edwards et al. (62)	Single-cue auditory delay EBC	EMG electrodes
Bolbecker et al. (63)	Single-cue auditory delay EBC	EMG electrodes
Bolbecker et al. (64)	ISI-shift single-cue auditory delay EBC	EMG electrodes
Forsyth et al. (65)	Single-cue auditory delay EBC	EMG electrodes
Parker et al. (66)	Single-cue auditory delay EBC	Infrared photo beam
Bolbecker et al. (67)	Single-cue auditory delay EBC	EMG electrodes
Coesmans et al. (68)	Single-cue auditory delay EBC	Magnetic distance measurement technique (i.e., measures distance between upper eyelid and lower eyelid)

**Table 3:** **EBC stimulus properties for studies of EBC in schizophrenia**.

Study		CS properties								US properties		
	Auditory					Visual				US intensity (psi)	US intensity measurementlocation	Dur. (ms)
	Modality	Freq. (Hz)	Intensity	CS intensity measurement	Dur. (ms)	Modality	Intensity	CS intensity measurement	Dur. (ms)			
Taylor and Spence (54)	–	–	–	–	–	6 cm milk glass disk	24	Apparent foot-candles	520	30 mmHg = 0.58 psi	Mercury manometer	–
O’Connor and Rawnsley (55)	Tone	1100	65	dB above threshold for each subject	800	–	–	–	–	65 mmHg = 1.26 psi	N/A	500
Spain (56)												
Auditory	Tone	1000	60	“Decibels in loudness”	1000	–	–	–	–	1 g	Measured at eye	160
Visual	–	–	–	–	–	Milk glass disk	700	Millilamberts	1000	1 g	Measured at eye	160
Sears et al. (57)	Tone	1000	75	dB	500	–	–	–	–	5	N/A	100
Hofer et al. (58)	Tone	1000	65	dB SPL	800	–	–	–	–	4	N/A	80
Stevens et al. (59)	Tone	300	80	dB SPL	800	–	–	–	–	2 bar = 29 psi	N/A	100
Marenco et al. (60)												
Trace	Tone	1000	80	dB	500	–	–	–	–	Between 5 and 6	N/A	100
Delay	Tone	1000	80	dB	500	–	–	–	–	Between 5 and 6	N/A	100
Brown et al. (61)	Tone	1000	80	dB SPL	400	–	–	–	–	10	At the source	50
Edwards et al. (62)	Tone	1000	80	dB SPL	400	–	–	–	–	10	At the source	50
Bolbecker et al. (63)	Tone	1000	80	dB SPL	400	–	–	–	–	10	At the source	50
Bolbecker et al. (64)	Tone	1000	80	dB SPL	300; 400; 600; 900	–	–	–	–	10	At the source	50
Forsyth et al. (65)	Tone	1000	80	dB SPL	400	–	–	–	–	10	At the source	50
Parker et al. (66)	Tone	1000	75	dB	500	–	–	–	–	5	N/A	100
Bolbecker et al. (67)	Tone	1000	80	dB SPL	400	–	–	–	–	10	At the source	50
Coesmans et al. (68)	Tone	650	75	dB	520	–	–	–	–	Adapted to minimum intensity required to reliably evoke a UR	N/A	20

**Table 4:** **EBC experiment and analysis parameters for studies of EBC in schizophrenia**.

Study	Experiment parameters								Dependent variable quantification criteria	
	ISI (ms)	Mean ITI (s)	Number of blocks	Trialsper block	Total trials(no. ofblocks)	Total no. paired trials	Pre-conditioning trials	Extinction trials	CR criterion (amplitude)	CR window (post-CS latency ms)
Taylor and Spence (54)	470	20	1	80	80 (1)	80	3 CS-alone, 1 US-alone	–	≥1 mm deflection of eyelid closure movement on polygraph (70)	200–470
O’Connor and Rawnsley (55)	350	Random b/t 20 and 40	1	48	48 (1)	30	3 CS-alone, 3 US-alone, 3 CS-alone	–	On CS-alone trials (*n* = 18) only: response amplitude 150% of maximum baseline amplitude (when cue light turned on before trial, 3–7 s pre-CS)	0–1.25 s post-CS onset for CS-alone test trials only
Spain (56)										
Auditory	500	20 ms	1	50	50 (1)	50	–	–	Non-voluntary eyelid movement	200–500
Visual	500	20 ms	1	50	50 (1)	50	–	–	Non-voluntary eyelid movement	200–500
Sears et al. (57)	400	12	1	70	70 (1)	63	10 US-alone trials	40 unpaired CS or US trials	Amplitude exceeds 10% of baseline UR amplitude (measured from 10 US-alone trials pre-conditioning) for paired and CS-alone trials	200–400
Hofer et al. (58)	720	12	6	12	72 (6)	48	–	–	Change in the curve of eyelid data waveform exceeds 0.4 cm for at least 30 ms on S+ and S− trials (71)	390–720
Stevens et al. (59)	700	1100 ms	1	70	70 (1)	–	–	–	–	Eyeblink response must occur 200–700 post-CS onset on S+ and S− trials
Marenco et al. (60)										
Trace	1540	18	7	11	77 (7)	70	10 CS-alone and 10 US-alone randomly presented	10 CS-alone and 10 US-alone randomly presented	Eyelid movement ≥0.5 mm for paired and CS-alone trials	150–1540
Delay	440	18	7	11	77 (7)	70	10 CS-alone and 10 US-alone randomly presented	10 CS-alone and 10 US-alone randomly presented	Eyelid movement ≥0.5 mm for paired and CS-alone trials	150–440
Brown et al. (61)	350	15	10	10	100 (10)	90	8 US-alone trials	20 CS-alone and 20 US-alone randomly presented	EMG amplitude over 5 SDs above pre-CS 125 ms baseline for each paired trial	100–350
Edwards et al. (62)	350	15	10	10	100 (10)	90	8 US-alone trials	–	EMG amplitude over 5 SDs above pre-CS 225 ms baseline for each paired trial	100–350
Bolbecker et al. (63)	350	15	10	10	100 (10)	90	8 US-alone trials	25 CS-alone and 25 US-alone randomly presented	EMG amplitude over 5 SDs above pre-CS 125 ms baseline for each paired trial	100–350
Bolbecker et al. (64)	250; 350; 550; 850	15	For each ISI: 5 (total = 10)	20	For each ISI: 100 (5) [total = 200 (10)]	For each ISI: 90 (total = 180)	8 US-alone trials	–	EMG amplitude over 5 SDs above pre-CS 125 ms baseline for each paired trial	150 pre-US onset
Forsyth et al. (65)	350	15	10	10	100 (10)	90	8 US-alone trials	–	EMG amplitude over 5 SDs above pre-CS 125 ms baseline for each paired trial	100–350
Parker et al. (66)	400	12	3	15	45 (3)	45	10 (5 CS-alone trials and 5 US-alone trials)	3 blocks of 30 extinction trials, each consisting of 20 CS-alone trials followed by 5 CS-alone and 5 US-alone trials presented pseudorandomly	Eyeblink amplitude exceeding 10% of baseline UR amplitude (measured during pseudoconditioning US-alone trials)	200–400
Bolbecker et al. (67)	350	15	10	10	100 (10)	90	8 US-alone trials	–	EMG amplitude over 5 SDs above pre-CS 125 ms baseline for each paired trial	100–350
Coesmans et al. (68)	500	Random intervals b/t 20 and 30	10	8	80 (10)	60	–	–	For paired and CS-alone trials, maximum eyelid closure occurring between 100 and 800 ms post-CS onset was first calculated. Blinks then classified as CRs if blink onset occurred within CR window (72)	150–525

**Table 5:** **Summary of main findings from studies of EBC in schizophrenia**.

Study	Summary of major findings
Taylor and Spence (54)	*CR* – Trend for increased percent visual CRs in “psychotics” vs. “neurotics.”
O’Connor and Rawnsley (55)	*CR –* No significant difference in number of CRs in response to CS-alone trials between groups (chronic paranoid SZ, chronic non-paranoid SZ, control).
	*Extinction –* Chronic paranoid SZ had significantly smaller “extinction scores” than controls.
Spain (56)	
Auditory	*CR –* Increased overall number of CRs in SZ vs. control, but effect not significant when examining subgroups matched for skin potential. SZ had significantly more visual than auditory CRs; opposite relationship in HNs. CRs for auditory EBC fewer in SZ vs. HN (but no statistical test reported).
Visual	*CR –* Increased overall number of CRs in SZ vs. control, but effect not significant when examining subgroups matched for skin potential. SZ had significantly more visual than auditory CRs; opposite relationship in HNs. CRs for visual EBC greater in SZ vs. HN (but no statistical test reported).
Sears et al. (57)	*CR* – SZ had significantly higher %CRs than controls and reached 70% CR learning criterion significantly faster (i.e., earlier in the experiment) than controls. Significantly shorter onset latency of all blinks in SZ vs. controls during paired trials (however, difference is not significant when group differences in conditioning level were accounted for and for CS-alone trials). CR amplitude significantly increased in SZ vs. controls in CS-alone trials.
	*UR –* Significantly longer UR latency in SZ vs. controls on US-alone trials.
Hofer et al. (58)	*CR –* Trend for controls to develop first CR before SZ. No significant difference between S+ and S− in SZ; there was a significant difference in controls for increased CRs to S+ vs. S−. Significantly greater %CRs in controls vs. SZ for S+ but no significant difference for S−. Significant group x reinforcement type (S+ or S−) x block interaction indicated controls showed increased %CRs in response to S+ as the experiment progressed.
Stevens et al. (59)	*CR –* No significant differences between groups in number of trials to reach learning “criterion” (i.e., 5 consecutive trials with an eyeblink response <500 ms pre-US onset to S+ but not S−).
Marenco et al. (60)	
Trace	*CR –* Analysis using the entire CR window appeared to be contaminated by spontaneous blinks (especially in SZ). A second analysis examining when in the CR window responses occurred revealed that SZ demonstrated increased early conditioned responses vs. controls, and slightly fewer later responses vs. controls. Frequency of early responses did not increase over time for SZ; control participants demonstrated trend-level increases in early responses over time. No significant effects when examining the last 500 ms as the CR window.
Delay	*CR –* No group differences in %CRs. Longer CR onset and peak latency for SZ vs. controls in “conditioners” during paired trials and CS-alone trials. More efficient “workratio” (a measure of CR efficiency of closing the eye at the time of US onset) in SZ vs. control “conditioners” during paired trials and CS-alone trials.
	*UR –* %URs significantly lower in SZ vs. controls in entire sample during paired trials. UR amplitude did not decrease across blocks in SZ vs. control “conditioners” during paired trials. For CS-alone trials, %UR-range responses significantly decreased in SZ vs. controls for entire sample (even larger effect when examining “conditioners” only).
Brown et al. (61)	*CR –* Significantly fewer %CRs overall in SZ vs. controls, and a trend for controls acquiring more CRs over time than SZ. Significantly shorter CR onset and peak latency in SZ vs. controls. Controls demonstrated decreased CR onset variability over time; SZ did not.
	*UR* – Trend for longer UR peak latency in SZ vs. control. *Extinction –* Significantly shorter CR onset and peak latency for SZ vs. controls.
Edwards et al. (62)	*CR –* Marginally significant difference between groups in learning, as indexed by the difference between mean %CRs in the last two blocks and mean %CRs in the first two blocks. Significantly higher %CRs in controls vs. SZ in block 9 of conditioning.
	*UR –* No significant differences in UR peak amplitude for paired or unpaired trials. No significant correlation between unpaired UR peak amplitude and mean CR amplitude.
Bolbecker et al. (63)	*CR –* Significantly decreased %CRs and shorter CR peak latency in SZ vs. controls.
	*UR –* Significantly slower UR peak latency in SZ vs. controls during paired trials. Significantly higher UR peak amplitude in SZ vs. controls for paired and unpaired trials.
	*Extinction –* Trend for fewer CRs during extinction for SZ vs. controls.
Bolbecker et al. (64)	*CR –* Decreased %CRs in SZ vs. controls across ISIs and later (i.e., closer to US) CR onset latency in SZ vs. controls across ISIs.
	*UR –* Significantly shorter UR latency in controls vs. SZ when first ISI presentation examined only (effect not significant when both first and second ISI presentations are considered).
Forsyth et al. (65)	*CR –* Decreased %CRs in SZ and SPD vs. controls, specifically in later blocks of conditioning. Trend for shorter CR peak latency in SZ and SPD vs. controls. CR amplitudes larger in a few later blocks in controls vs. SPD and SZ.
	*UR –* Significantly higher UR peak amplitude in SZ vs. controls and SPD.
Parker et al. (66)	*CR –* Significantly greater %CRs in controls compared to SZ in middle and late phases of conditioning. CR peak latency significantly shorter in SZ in middle phase of conditioning.
Bolbecker et al. (67)	*CR –* Significantly lower rate of learning in SZ and relatives compared to controls. Controls increase in %CRs over time more than relatives and SZ.
	*UR –* Larger UR amplitude during paired trials only in SZ vs. controls.
Coesmans et al. (68)	*CR –* Significantly fewer %CRs in SZ compared to controls, with a trend-level group x block interaction. Controls demonstrated significantly higher learning index (defined as the difference in first and last block number of CRs) vs. SZ.

*SZ, individuals with schizophrenia; HN, healthy non-psychiatric controls; SPD, individuals with schizotypal personality disorder*.

Various domains of information from these 15 studies examining EBC in the schizophrenia spectrum were then recorded and organized, including sample characteristics (see Table 1), parametric properties of the EBC tasks and analyses, and major findings (see Tables 2–5). In the review of this literature, careful attention was paid to (1) findings that occur consistently across studies and across research groups, (2) the relationship of medication status to consistent findings, (3) any sample characteristics or parametric variability (in either EBC paradigms or analyses) that may contribute to heterogeneity of findings, (4) correlates of EBC performance in individuals along the schizophrenia spectrum, and (5) the implications of the findings of this review for current systems-level and neurobiological theories of schizophrenia.

## Results

### Conditioning

#### Conditioned Responding (e.g., %CRs)

Of the 15 studies of delay EBC in schizophrenia, 9 demonstrated decreased CRs compared to controls (58, 61–68), 4 found no group differences in rates of conditioned responding (54, 55, 59, 60), and 2 reported facilitated conditioning in schizophrenia (56, 57). It should be noted, however, in one study (56) which reported overall increased percent CRs in schizophrenia vs. controls, that when the auditory and visual EBC results are considered separately, schizophrenia patients yielded fewer CRs when the CS was an auditory vs. visual stimulus.

#### CR Onset Latency

One study reported shorter CR onset latencies in individuals with schizophrenia vs. controls (61). Two studies reported longer CR onset latencies in schizophrenia vs. controls (60, 64). Two studies reported no significant differences between groups (66, 67). One study reported blink onset latency results regardless of CR or UR performance, and therefore cannot be considered with either CR or UR results [see Ref. (57) in Table 5 for these and CS-alone latency findings].

#### CR Peak Latency

Three studies reported shorter peak latency in individuals with schizophrenia vs. controls (61, 63, 66). One study reported longer CR peak latency in schizophrenia vs. controls (60), and three studies reported no significant differences between groups (62, 64, 65).

#### CR Amplitude

Five studies reported no significant differences between groups for CR peak amplitude (60, 61, 63, 66, 67). Sears and colleagues (57) reported increased CR amplitude in individuals with schizophrenia vs. controls in CS-alone trials. In *post hoc* analyses of individual blocks, Forsyth and colleagues (65) found increased CR amplitudes in controls vs. schizophrenia and SPD in later but not earlier blocks of conditioning.

### Medication Effects

Of the 15 published studies, 13 reported medication status and all but one of these (56) included information specific to antipsychotic medication status. In 10 of these 12 studies, most participants in the schizophrenia sample were currently taking antipsychotic medication. In terms of conditioning effects, 8 of these 10 studies of medicated individuals reported decreased conditioning (e.g., decreased percent CRs) in individuals with schizophrenia compared to controls (58, 61–65, 67, 68). In the other two studies of medicated individuals, no group differences in conditioning rates were found (59, 60).

In 2 of the 12 studies, the entire schizophrenia group was antipsychotic-free for 3 weeks (57, 66). Sears and colleagues (57) reported facilitated conditioning in these participants, whereas Parker and colleagues (66) reported impaired conditioning. In addition, 3 of the 12 studies analyzed data from antipsychotic-free subsamples of individuals with schizophrenia (63, 64, 68). When Bolbecker and colleagues (63) re-analyzed their data including only the medication-free subset of individuals with schizophrenia and their age-matched controls (with a sample size in each group of *n* = 13, similar to other stand-alone studies of antipsychotic-free schizophrenia), they found decreased CRs and shorter CR peak latencies in these individuals with schizophrenia – with even larger effect sizes than in the full sample of individuals with schizophrenia. The authors reported no significant correlations between EBC dependent variables and chlorpromazine equivalent dosages (63), as did Brown and colleagues (61). Similarly, in a later study, Bolbecker and colleagues (64) reported no significant differences between schizophrenia participants medicated with antipsychotics vs. those who were medication-free. Finally, Coesmans and colleagues (68) reported no effect of group on percent CRs or “learning index” (change in number of CRs from first to last conditioning block) when comparing the three subgroups of individuals with schizophrenia (those taking atypical antipsychotics, typical antipsychotics, and those who were antipsychotic medication-free), and no significant correlation between learning index and chlorpromazine equivalent dosages.

Finally, both studies including intermediate schizophrenia spectrum participants [individuals with SPD (65) and first-degree relatives (67)] reported that there was no antipsychotic use in either of these populations. In these studies both individuals with SPD and first-degree relatives of individuals with schizophrenia were impaired in EBC.

### Unconditioned Responses

UR measures on paired trials are reported less frequently in the literature. With regard to percentage of URs, one study reported decreased percent URs in individuals with schizophrenia vs. controls (60). With regard to UR latency, two studies reported slower UR peak latency in individuals with schizophrenia vs. controls (63, 64), while three other studies reported no significant differences between groups (61, 65, 67). Finally, with regard to UR amplitude, three studies reported increased UR amplitude in schizophrenia vs. controls (63, 65, 67), whereas three studies reported no significant group difference (61, 62, 64). And, one study reported a significant group by block interaction showing consistently diminished UR amplitude in individuals with schizophrenia compared to controls, and larger initial UR amplitude in controls that decreased across blocks (60).

Importantly, several studies explored group differences in URs to unpaired unconditioned stimuli during pre-conditioning trials or pseudoconditioning (prior to paired trial presentation). Such pre-conditioning measures test for pre-existing differences between groups in the ability to generate a blink in the absence of recent associatively salient stimuli and habituation. Marenco and colleagues (60) reported no group differences in baseline UR activity; Edwards and colleagues (62) reported no group difference in baseline UR amplitude. Bolbecker and colleagues reported no group differences in UR peak amplitude or latency in individuals with schizophrenia compared to controls in one article (67) and increased UR amplitude in another (63) – in both cases suggesting that conditioning deficits could not be accounted for by pre-existing group differences in eyeblink responses. However, Sears and colleagues (57) reported longer UR latency in individuals with schizophrenia compared to controls for US-alone trials.

### Extinction

Four studies reported no significant differences between extinction rate in individuals with schizophrenia and controls (60, 61, 63, 66). However, interpretation of this finding is complicated by the group differences in percent CRs during the acquisition phase reported by three of the studies (61, 63, 66). Finally, Brown and colleagues (61) reported shorter CR onset and peak latency in individuals with schizophrenia vs. controls during extinction.

### Spontaneous Blink Rate

Several studies excluded individual trials in which a blink occurred at a time during a trial that would render CR production impossible (i.e., immediately prior to CS onset) [Ref. (61–65, 67); see Ref. (60) for a more liberal window for trial exclusion]. Most of these studies also reported no significant group differences in this rough estimate of spontaneous blink rate [Ref. (60, 63–65, 67), but see Ref. (61)].

### Alpha Responses

Three studies examined group differences in alpha responses, which are reflexive orienting responses to the tone (importantly, alpha responses are non-associative). All three studies reported no group differences in the rate of alpha responses (57, 58, 60). Marenco and colleagues (60) reported earlier onset of the alpha response in controls vs. individuals with schizophrenia.

### EBC Correlates

#### Symptoms and Demographic Variables

Multiple studies have failed to find significant relationships between schizophrenia symptom severity and EBC dependent variables (61, 63, 68). Brown and colleagues (61) and Bolbecker and colleagues (63) also reported null results between symptom severity and extinction dependent variables. Parker and colleagues (66) found no significant correlations between positive or negative symptoms and the three phases of conditioning the authors used to analyze their EBC data (i.e., early, middle, and late); however, negative symptoms were significantly correlated with late-phase extinction of the CR. In the earliest examination of symptom correlates of EBC, O’Connor and Rawnsley (55) reported no significant correlation between EBC and introversion scores [but see Spain (56) for EBC correlates of clinician-rated withdrawal]. Finally, in their investigation of demographic correlates of EBC, Coesmans and colleagues (68) also reported non-significant correlations between learning index and age and years of education.

#### Neuropsychological Variables

Bolbecker and colleagues (63) reported significant positive correlations between average percent CRs and both WASI IQ estimates and the WASI Vocabulary subscale in controls, but not in individuals with schizophrenia. The Matrix Reasoning subscale was not significantly correlated with average percent CRs in either group. Forsyth and colleagues (65) reported a significant positive correlation between percent CRs and Digit Symbol score (a subscale of the WAIS) for schizophrenia spectrum participants (i.e., individuals with schizophrenia and SPD were combined into one group). This significant correlation held when individuals with schizophrenia were analyzed separately, but not when individuals with SPD were analyzed separately. Additionally, the authors reported no significant correlations between Digit Symbol score and percent CRs in controls, or between percent CRs and the Picture Completion, Similarities, or Digit Span WAIS subscales in either controls or schizophrenia spectrum participants (65). Using aggregate cognitive domain scores from a battery of neuropsychological tests in patients, Parker and colleagues (66) reported a significant positive relationship between both aggregate language and motor scores and CR timing during early conditioning; motor scores were also correlated with middle-phase extinction of the CR. Finally, Coesmans and colleagues (68) reported a significant positive correlation between EBC learning index and saccade adaptation strength in controls, but not individuals with schizophrenia, while no significant correlations were found in either group between EBC learning index and saccade adaptation speed.

#### Neuroimaging Measures

In a study of cerebellar volumetric correlates of EBC, Edwards and colleagues (62) reported a significant positive correlation between anterior lobe volume and CR onset latency, and a significant negative correlation between anterior lobe volume and UR amplitude (in response to paired trials) in controls, but no significant correlations between cerebellar MRI volume and EBC dependent variables in individuals with schizophrenia. Parker and colleagues (66) analyzed PET data according to phases of conditioning (i.e., early, middle, and late), and reported decreased rCBF in individuals with schizophrenia compared to controls in frontal, thalamic, and cerebellar regions during both acquisition and extinction (among other loci). In summarizing findings of hypofrontality during EBC, the authors highlighted decreased rCBF in individuals with schizophrenia compared to controls in the contralateral medial frontal gyrus during all phases of conditioning, and the contralateral middle frontal gyrus during the early and middle phases of conditioning. The authors also highlighted decreased rCBF in contralateral cerebellar lobules IV and V in individuals with schizophrenia compared to controls during all phases of conditioning, with a group difference in ipsilateral cerebellar lobule VI during late acquisition only. Finally, group differences in rCBF in the thalamus were significant during early and late conditioning. Regarding rCBF during extinction, the authors highlighted decreased rCBF in individuals with schizophrenia compared to controls during all phases of extinction in the medial and middle frontal gyri and in cerebellar lobule IX. Additional loci of decreased cerebellar rCBF in individuals with schizophrenia compared to controls included cerebellar lobules IV and V during middle extinction, and cerebellar lobules IV, V, and VI during late extinction. Finally, the authors highlighted that decreased thalamic rCBF in individuals with schizophrenia was significant during early phase extinction (66).

## Discussion

### Conditioning (i.e., %CRs)

In reviewing the literature investigating delay EBC in schizophrenia, decreased percent conditioned responses in individuals with schizophrenia compared to non-psychiatric controls emerges as the single consistent, robust, and replicated finding. Diminished conditioning in schizophrenia is highly suggestive of cerebellar dysfunction, given the crucial role of the cerebellum in the circuit underlying delay EBC. Moreover, as discussed in the following paragraphs, there are no extraneous variables (i.e., medication status, sample size, different analytical approaches, parametric variability, non-associative blinking function, and investigative group) that could fully account for these EBC deficits in schizophrenia.

In investigating the possible driving role of medication in the observed EBC deficits (i.e., decreased %CRs) in individuals with schizophrenia, it is crucial to note that both medicated and non-medicated samples demonstrate conditioning deficits in individuals with schizophrenia (see Medication Effects subsection of Section “RESULTS”). Also important to this question of the effect of antipsychotic medication on EBC are the findings of EBC deficits in a non-medicated subsample (63), as well as the failure to find group differences in medicated vs. unmedicated individuals with schizophrenia (64, 68).

However, it is important to note that even the “medication-free” samples and subsamples reported above are not medication-naïve samples. While a small number of participants in the most recent studies [*n* = 5 in Parker et al. (66), and *n* = 6 in Coesmans et al. (68)] were naïve to antipsychotics, the small sizes of these groups precluded meaningful analyses investigating the effect of antipsychotic-naïve medication status. Therefore, while it appears unlikely based on the current review that recent use of antipsychotic medication drives EBC deficits, it is impossible to rule-out the long-term effects of antipsychotic use in individuals with schizophrenia in the results of the study of “medication-free” samples and subsamples.

Eyeblink conditioning studies of intermediate genotypes and phenotypes of schizophrenia such as first-degree relatives (67) and SPD (65) that have demonstrated conditioning deficits in these groups are very important, especially given the absence of studies using medication-naïve or first-episode schizophrenia groups. Neither of these study groups were taking antipsychotic medication. This suggests that EBC deficits are related to the genetic/biological pathophysiology of schizophrenia, not the history of or current antipsychotic medication use.

In addition to medication status, examination of Tables 1–5 reveals no systematic sample characteristic, parameter, or analytic approach that could be driving this review’s main finding of EBC deficits in schizophrenia. Indeed, EBC deficits occur across samples of varying ages and gender composition, and in studies using a range of EBC stimulus parameters and experimental design (e.g., CS/US duration, ISI, ITI, and pre-conditioning trials or pseudoconditioning) and analysis (e.g., CR window and criterion) specifications. Furthermore, potentially confounding issues such as spontaneous blink rate and baseline blinking function have been investigated by several groups, with no convincing evidence that these variables bias EBC experimental results.

Furthermore, it appears as though many studies reporting null findings or facilitated conditioning may have parametric or analytic variations that could account for such results. Specifically, Taylor and Spence (54) used a visual delay EBC paradigm, and the diagnostic criteria for the disorder differed substantially from those used in recent decades. Furthermore, the idiosyncratic analytic approaches of other studies may account for the reported null findings. For example, rather than quantifying rate of conditioning, Stevens and colleagues (59) measured the number of trials it took for participants to reach “criterion,” or five consecutive CRs. This style of analysis is not reported in most other studies. Another study appeared to restrict their analysis such that relatively less data are included compared to other studies. Specifically, O’Connor and Rawnsley (55) only used 18 unpaired CS-alone trials to measure conditioning, rather than attempting to detect CRs across all paired trials over the course of conditioning. Finally, Sears and colleagues (57) did not include a measure of spontaneous blink rate; it is therefore possible that group differences in non-associative blinking could have confounded the reported findings of facilitated conditioning in schizophrenia. More research is necessary to determine whether these varied findings are due to these methodological differences or, in fact, reflect inconsistencies in EBC deficits in schizophrenia across studies.

### CR Timing

Group differences in timing of the conditioned response (i.e., onset and peak latency) have been reported far less frequently than rate of conditioning (i.e., percent CRs). Among studies reporting these variables, there is inconsistency in how onset latency is calculated and whether the algorithm used to calculate onset latency is reported. Results are also inconsistent, with findings reported in both directions and null results. However, the proportion of findings reporting some group difference (*N* = 7) in CR timing vs. null results (*N* = 6) suggests that there may be abnormalities in the timing of the conditioned response in individuals with schizophrenia.

### Interpretation of Correlate Findings

Parker and colleagues’ (66) findings of both impaired conditioning and decreased cerebellar blood flow in individuals with schizophrenia compared to controls during delay EBC strongly suggest that cerebellar neural dysfunction underlies the behavioral EBC abnormalities consistently reported in individuals with schizophrenia. This is a crucial piece of evidence, as authors reporting previous findings of impaired delay EBC in individuals with schizophrenia have inferred underlying cerebellar dysfunction given the well-established delay EBC cerebellar circuitry in non-human animals.

In addition, EBC correlates of neuropsychological performance are reported by a few studies (63, 65, 66). This shared variance between cerebellar-dependent EBC performance and cognition indicates that the cerebellum may be a shared neural substrate between these two processes, which is consistent with cerebellar involvement in cognitive as well as motor function.

### Limitations and Future Directions

One critical conclusion from this review is that antipsychotic medications do not appear to be driving the EBC deficit observed consistently in schizophrenia. However, this conclusion is primarily based on the study of EBC in unmedicated (rather than never-medicated) individuals with schizophrenia, first-degree relatives and individuals with schizotypal personality disorder. While the robustness of the EBC deficit in these populations is obviously compelling, a logical and important next step is conducting delay EBC in first episode and/or never-medicated individuals. Second, significant variability in methodological and analytic strategies across EBC studies precluded a meta-analytic approach; therefore, as more studies are conducted using consistent methods, statistical analyses, and reporting, this approach should be considered. Third, further replication of the main findings of this review article (i.e., an EBC deficit in schizophrenia) is essential given that one investigative group has accounted for most patients studied (6 of 15 studies).

Finally, further work investigating the neural activity in the cerebellum during delay EBC in schizophrenia is essential to elucidating the specific contribution of the cerebellum in driving impairments in delay EBC. Specifically, the fine-grained spatial resolution of fMRI could prove essential to understanding which regions of the cerebellum underlie delay EBC in humans, and where this circuit is degraded in schizophrenia.

### EBC Findings Within the Context of Theories of Schizophrenia

Overall, the reported deficits in cerebellar-dependent EBC in individuals with schizophrenia are consistent with the theory of cognitive dysmetria, in which the cerebellum is one node in a circuit regulating the fluid temporal coordination of motor, cognitive, and affective information, the disruption of which is hypothesized to be a common underlying precursor to the heterogeneous downstream expressions of the phenomenology of schizophrenia (29, 73). The cerebellum is believed to play a unique role in this circuit mediating the coordination (or instantiating the discoordination) of mental activity, which also includes the prefrontal cortex and the thalamus. Specifically, it is the feedback (via the thalamus) between the prefrontal cortex (and the higher-order cognitive processes instantiated therein) and the cerebellum (which is notable for cytoarchitecture conducive to large-scale parallel processing and its role in coordination, sequencing, and timing) that is hypothesized to instantiate the fluid temporal coordination of mental activity (29, 73). As stated above, consistent deficits in performance on one of the most robust and well-understood (with respect to underlying circuitry) cerebellar tasks in individuals with schizophrenia provide evidence consistent with the theory of cognitive dysmetria, and this finding is germane to its arguably most critical node [Andreasen (29) initially identified assays of cerebellar function, specifically citing EBC as a potential example, as the litmus test through which the theory can be falsified]. In addition, the relationship between cerebellar function and cognitive function supports this theory (63, 65, 66).

More specifically, the possible mechanisms of the cerebellum’s contribution to higher-order cognitive function have been hypothesized to parallel that proposed by control theory in the domain of motor control [see Ref. (74, 75), for review]. The cerebellum is hypothesized to contribute to the coordination of movement via internal models (both forward and reverse) (74), which are neural representations that can be trained to simulate the dynamics of motor action (74–76). Forward models are believed to receive input that duplicates the motor command (termed an efference copy) sent by the motor cortex (which controls movement) after the motor cortex receives a higher-order instructor command (i.e., from the premotor cortex), and output a prediction of what the sensory consequences of that command will be (a corollary discharge). The forward model is tuned by a mechanism that compares sensory predictions of the model to actual sensory input, which has been hypothesized to occur in the inferior olive. Once a forward model is adequately trained, it can provide useful feedback (via the thalamus) to the primary motor cortex, which executes motor commands (74, 75). An inverse model, conversely, can eventually conduct feed-forward motor control in response to a higher-order instructor command. While error-related feedback processing in the inferior olive is also hypothesized to tune inverse models, inverse models are trained by comparing motor output to the initial instructor command, and this feedback is mediated through the motor cortex (74).

In generalizing the function of internal models in the cerebellum to a role in cognition, it has been proposed that there are areas in the prefrontal cortex (following a higher-order instructor command, as in the example using motor function) that send commands to areas of the cortex that instantiate psychological processes and manipulate these areas in much the same way the motor cortex manipulates the motor system. In this way, the cerebellum receives an efference copy of this command and can learn and execute forward models that would simulate processing in the target brain area and provide feedback to the prefrontal cortex (74, 75). Using an inverse model, the cerebellum could actually perform feed-forward control of cognitive function (again following an instructor signal) by acting directly on the target brain area (74).

These putative mechanisms of cerebellar contributions to cognition are supported by the frequently cited uniformity of cerebellar cytoarchitecture, which, along with its circuitry suggest that the cerebellum is performing a uniform process across a variety of cortical inputs (74, 75). In addition, the matched increase in both cerebral and cerebellar neurons in humans as well as high connectivity between the cortex and cerebellum also indicate the proposed mechanisms of cerebellar contributions to cognition are physiologically plausible (77, 78). Finally, translational evidence in support of cerebellar contributions to cognition can be found in comparing cortical projections to the cerebellum in humans and macaque monkeys, where the largest proportion of the projections in humans originates in prefrontal cortex vs. motor areas in macaque monkeys [see Ref. (75) for review]. In light of this physiological and translational evidence, the proposed function of internal models as a mechanism of cerebellar contributions to cognition seems both anatomically and evolutionarily sound. Ramnani (75) has further described internal models as ideally suited to rapid, highly accurate, efficient processing of routine, well-practiced cognitive processes, whereas cortical mechanisms are best suited for flexible though less efficient processing, which would be important for processing novel problems or generalizing cognitive processes across different contexts.

In addition to being a robust assay of cerebellar function, EBC is especially germane to the putative mechanisms outlined above in light of the proposed mechanism of error correction of internal models. Specifically, the feedback-related tuning of internal models is believed to be instantiated through error signals sent from climbing fibers (originating in the inferior olive), which results in LTD at the parallel fiber–Purkinje cell synapse when climbing and parallel fibers are simultaneously activated (74). In EBC, US information is transmitted through climbing fibers from the inferior olive, and is often conceptualized as an error signal, and an identical LTD mechanism as that described above is believed to be an integral part of cerebellar cortical plasticity during conditioning [see Ref. (79) for review]. It has previously been suggested that dysfunctional internal models may be the mechanism of cerebellar-mediated cognitive and affective dysfunction in schizophrenia (22, 74). It is therefore notable that the findings of this review indicating deficits in cerebellar function in schizophrenia, and more importantly EBC deficits specifically, may be indicative of dysfunctional cerebellar internal models, which may be mediating the cardinal cognitive and affective symptoms of the disorder.

However, neuropsychological correlates of EBC in individuals with schizophrenia have been rarely investigated. Furthermore, the consistently reported non-significant correlations between delay EBC and symptom severity in individuals with schizophrenia is surprising given the putative role of the cerebellum in the pathophysiology of schizophrenia. It is possible that the contributions of cerebellar deficits to the pathological processes of schizophrenia are more proximal effects on timing and coordination of information, whereas symptoms and impaired neuropsychological function are more distal manifestations of the disorder that are affected by many factors and are not linearly related in magnitude to cerebellar dysfunction. Restricted range in neuropsychological and symptom measures and/or floor effects might also obscure any systematic relationships between these variables and EBC performance. Finally, symptoms are a state-dependent variable; the potentially transient and fluctuating nature of symptom severity might also account for the lack of reported correlates. Alternatively, it is possible that cerebellar dysfunction in areas outside of the delay EBC circuitry is related to symptom severity and neuropsychological function. Still, more research is necessary to understand the relationships between cerebellar-mediated dysfunction and cognitive and clinical variables.

Importantly, EBC performance deficits in schizophrenia may have implications for glutamatergic models of the disorder given that glutamate is the primary excitatory neurotransmitter in the cerebellum [see Ref. (80) for review]. The glutamate model of schizophrenia hypothesizes dysfunction of the NMDA type of glutamate receptor [see Ref. (81) for overview]. Non-human animal research has implicated NMDA receptors in the interpositus nucleus in CR acquisition [Ref. (82); see Ref. (79) for a thorough review of the neural mechanisms of EBC]. Given that the “memory trace” of delay EBC has been localized to the anterior interpositus nucleus, it is therefore possible that impairments in conditioning in schizophrenia (reported most frequently as a decrease in percent CRs, or impaired CR acquisition) are related to NMDA receptor dysfunction in the interpositus nucleus in schizophrenia.

There is also substantial glutamatergic transmission in the cerebellar cortex; therefore, abnormalities in CR timing (largely mediated by the cerebellar cortex) may also be indicative of NMDA receptor dysfunction in schizophrenia. While NMDA receptors have been reported in the cerebellar cortex (80), they were traditionally not believed to play a role in the cellular mechanism (i.e., LTD at the parallel fiber-Purkinje cell synapse following both parallel and climbing fiber input to Purkinje cells) believed to underlie EBC-related learning in the cerebellar cortex [see Ref. (83) for review]. Importantly, however, there is more recent evidence that NMDA receptors at the climbing fiber–Purkinje cell synapse may in fact contribute to LTD at the parallel fiber–Purkinje cell synapse (84). Furthermore, more broad conceptualizations of the substrates of cerebellar learning are emerging that suggest that mechanisms of cerebellar cortical plasticity and neural activity beyond LTD at the parallel fiber-Purkinje cell synapse (some involving NMDA receptors) may be involved in EBC (85, 86). Accordingly, more research is necessary to determine the role of glutamate in reported EBC timing abnormalities in schizophrenia.

In addition to the glutamate hypothesis, abnormalities in the endocannabinoid system in schizophrenia [see Ref. (87) for brief review] are also implicated by the current review findings. Edwards and Skosnik (87) have proposed EBC neural circuitry including endocannabinoids as retrograde signals serving to neuromodulate cerebellar cortical activity, thereby influencing CR timing and morphology. It is therefore possible that CR timing abnormalities in schizophrenia are indicative of abnormalities in the endocannabinoid system [see Ref. (87) for discussion].

## Author Contributions

JK, WH, AB, and BO conceptualized the review article. JK conducted the review. JK and WH drafted the paper, and AB and BO provided critical review. All authors approved and agree to be accountable for the final version of the manuscript.

## Conflict of Interest Statement

The authors declare that the research was conducted in the absence of any commercial or financial relationships that could be construed as a potential conflict of interest.
